# Reconsidering the Sporulation Characteristics of Hypervirulent *Clostridium difficile* BI/NAP1/027

**DOI:** 10.1371/journal.pone.0024894

**Published:** 2011-09-15

**Authors:** David A. Burns, Daniela Heeg, Stephen T. Cartman, Nigel P. Minton

**Affiliations:** Clostridia Research Group, School of Molecular Medical Sciences, Centre for Biomolecular Sciences, University of Nottingham, Nottingham, United Kingdom; University of Connecticut, United States of America

## Abstract

*Clostridium difficile* is the leading cause of antibiotic-associated diarrhoea and a major burden to healthcare services worldwide. In recent years, *C. difficile* strains belonging to the BI/NAP1/027 type have become highly represented among clinical isolates. These so-called ‘hypervirulent’ strains are associated with outbreaks of increased disease severity, higher relapse rates and an expanded repertoire of antibiotic resistance. Spores, formed during sporulation, play a pivotal role in disease transmission and it has been suggested that BI/NAP1/027 strains are more prolific in terms of sporulation *in vitro* than ‘non-epidemic’ *C. difficile* types. Work in our laboratory has since provided credible evidence to the contrary suggesting that the strain-to-strain variation in *C. difficile* sporulation characteristics is not type-associated. However, the BI/NAP1/027 type is still widely stated to have an increased rate of sporulation. In this study, we analysed the sporulation rates of 53 *C. difficile* strains, the largest sample size used to-date in such a study, including 28 BI/NAP1/027 isolates. Our data confirm that significant variation exists in the rate at which different *C. difficile* strains form spores. However, we clearly show that the sporulation rate of the BI/NAP1/027 type was no higher than that of non-BI/NAP1/027 strains. In addition, we observed substantial variation in sporulation characteristics within the BI/NAP1/027 type. This work highlights the danger of assuming that all strains of one type behave similarly without studying adequate sample sizes. Furthermore, we stress the need for more rigorous experimental procedures in order to quantify *C. difficile* sporulation more accurately in the future.

## Introduction

*Clostridium difficile*, a Gram-positive, spore-forming bacterium, is the major underlying cause of antibiotic-associated diarrhoea. Outbreaks of *C. difficile* infection (CDI) have led to patient isolation, ward closures and, sometimes, hospital closure. In the United States of America alone, CDI is estimated to affect over 500,000 people each year and cost the healthcare system over $3 billion per year [1], [2]. Endospores, formed during sporulation, are able to resist a variety of industrial cleaning agents and can persist on surfaces in healthcare settings for prolonged periods of time [3], [4]. Following ingestion by susceptible individuals, spores return to vegetative cell growth through germination which allows for colonisation and production of the characteristic toxins [5]. Consequently, CDI can cause intestinal perforation, toxic megacolon and a potentially fatal pseudo-membranous colitis [6]. The spore form of *C. difficile* is, therefore, crucial for disease transmission.

The challenge of CDI has increased with the emergence of so-called ‘hypervirulent’ strains belonging to restriction endonuclease type BI, North American pulsed-field type 1 and PCR-ribotype 027 (BI/NAP1/027). Strains of the BI/NAP1/027 type have become highly represented among clinical isolates from recent outbreaks and are associated with an expanded repertoire of antibiotic resistance, more severe disease and higher relapse rates [7], [8]. Unsurprisingly, there is widespread interest in understanding the underlying factors that have led to the emergence of strains such as those of the BI/NAP1/027 type.

Some BI/NAP1/027 strains are believed to produce higher levels of toxin in the laboratory than strains belonging to other types [9] and a number of recent studies have concluded that strains of the BI/NAP1/027 type are also more prolific in terms of sporulation *in vitro* [10], [11], [12], [13], [14], [15]. However, work in our laboratory has since provided credible evidence to the contrary suggesting that the strain-to-strain variation in *C. difficile* sporulation characteristics is not type-associated [16], [17]. In spite of this evidence, the BI/NAP1/027 type is still widely stated to have an increased rate of sporulation [1], [18], [19], [20]. On analysis of the other studies currently in the literature, it is apparent that sample sizes have remained small and, perhaps most importantly, the methods used to quantify sporulation have severe limitations [21].

Previous studies have varied in the choice of growth medium, the time over which sporulation was measured and the procedures used for expressing sporulation rates [11], [12], [13], [14], [15]. For example, some studies have expressed sporulation as the ratio of spores to vegetative cells within the population [11], [12], [15]. However, this relative measure can be affected by growth differences among strains and also by the survival of non-sporulating vegetative cells. Another study enumerated spore titres solely based on colony-forming units (CFU) after ethanol treatment, a measure that cannot distinguish between sporulation, ethanol resistance, or spore germination and outgrowth [14]. Finally, a recent study expressed sporulation as the proportion of CFU recovered following exposure to aerobic conditions [10]. Unfortunately, there is no evidence present in the literature to-date describing whether exposure of *C. difficile* vegetative cells to oxygen has a bacteriostatic or bacteriocidal effect, calling into question the conclusions of the authors.

Our previous analysis of *C. difficile* sporulation included seven BI/NAP1/027 strains, including isolates from both North America or Europe to minimise the risk of sampling clonal strains [16]. The diversity we observed within one group of seven *C. difficile* strains suggests that, in order to accurately determine variation in sporulation between different groups, an appropriate sample size will need to be much larger than seven strains. However, while the sample used in our study was not particularly large, this did represent the largest sample size used to-date in such a study. Other reports of *C. difficile* sporulation rates have been limited to as few as one representative strain of each type [14], [15]. Consequently, the continued use of small sample sizes may be contributing to the conflicting evidence that is currently present in the literature.

In this study, we sought to clarify how the sporulation rates of *C. difficile* BI/NAP1/027 strains compare to isolates of other types. We analysed 53 strains of *C. difficile* isolated in the USA, Canada and Europe, including 28 BI/NAP1/027 and 25 non-BI/NAP1/027 strains (Table 1). Notably, this is the largest sample size used to date in a study on *C. difficile* sporulation. Substantial diversity was observed in the rate of *C. difficile* sporulation among isolates but, importantly, this diversity did not correlate to type. Crucially, the BI/NAP1/027 type was not found to have a higher sporulation capacity than strains of other types.

**Table 1:** strains used in this study.

Strain name	PCR-ribotype	Country	Source / Reference
**001-2**	001	The Netherlands	Ed Kuijper
**001-3**	001	The Netherlands	Ed Kuijper
**001-5**	001	The Netherlands	Ed Kuijper
**001-7**	001	The Netherlands	Ed Kuijper
**CD 16839**	002	Hungary	Ed Kuijper
**8083598**	002	The Netherlands	Ed Kuijper
**8085053**	002	The Netherlands	Ed Kuijper
**8092419**	002	The Netherlands	Ed Kuijper
**9001966**	002	The Netherlands	Ed Kuijper
**MF081988**	002	Ireland	Ed Kuijper
**VPI10463**	003		[26]
**675,1**	017	Romania	Ed Kuijper
**81568**	027	France	Ed Kuijper
**5108111**	027	The Netherlands	Ed Kuijper
**32219**	027	Luxembourg	Ed Kuijper
**M246**	027	Ireland	Ed Kuijper
**2191**	027	Ireland	Ed Kuijper
**51556**	027	Germany	Ed Kuijper
**60902**	027	Switzerland	Ed Kuijper
**26131**	027	Finland	Ed Kuijper
**CDC 32**	027	USA (historical)	[27]
**CDC 38**	027	USA	[27]
**M13042**	027	Canada	[27]
**DH349**	027	East of England, Cambridge, UK	Val Hall
**DH320**	027	NE England, Newcastle, UK	Val Hall
**DH1916**	027	SW England, Torbay, UK	Val Hall
**DH1396**	027	SE England, Slough, UK	Val Hall
**DH1432**	027	London, Barnet, UK	Val Hall
**DH1834**	027	East of England, Ipswich, UK	Val Hall
**DH1466**	027	East Midlands, Northampton, UK	Val Hall
**DH1751**	027	Yorkshire & Humberside, Bradford, UK	Val Hall
**027 Alexander**	027	Austria	Ed Kuijper
**DH1858**	027	NE England,Sunderland, UK	Vall Hall
**51557**	027	Germany	Ed Kuijper
**DH478**	027	SW England, Taunton, UK	Val Hall
**DH361**	027	London, Lewisham, UK	Val Hall
**DH131**	027	NW England, Manchester, UK	Val Hall
**R20352**	027	Canada	Val Hall
**R12087**	027	Historical EU strain (mid 80s)	Val Hall
**R20291**	027	Stoke Mandeville, UK	Jon Brazier
**Wilcox 078**	078	Leeds, UK	Mark Wilcox
**31662**	078	The Netherlands	Ed Kuijper
**2016**	078	Ireland	Ed Kuijper
**7004578**	078	The Netherlands	Ed Kuijper
**7009825**	078	The Netherlands	Ed Kuijper
**R9764**	081	Cardiff, UK	Jon Brazier/Ed Kuijper
**R10432**	106	Birmingham, UK	Val Hall
**R12801**	106	Bristol, UK	Val Hall
**R15347**	106	London, UK	Val Hall
**R22079**	106	Sheffield, UK	Val Hall
**R23942**	106	Cambridge, UK	Val Hall
**R25469**	106	Antrim, Northern Ireland	Val Hall
**R10459**	106	Cardiff, UK	Jon Brazier/Ed Kuijper

## Results

### Comparison of the growth rates of C. difficile isolates

**Figure 1:**
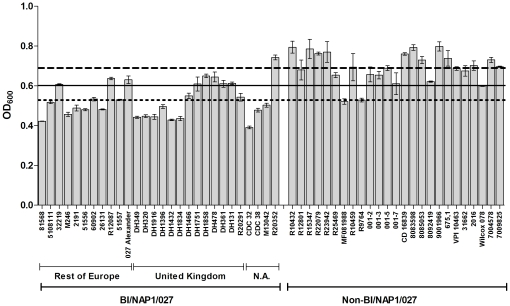
The cell density of *C. difficile* isolates in BHIS broth after 20 h measured as OD_600_. The BI/NAP1/027 strains were originally isolated from North America (N.A.), the UK, or the rest of Europe. The data represent the average of three independent experiments and error bars indicate standard errors of the means. The solid line represents the average OD_600_ of all 53 isolates at 20h, the dotted line represents the average OD_600_ of the BI/NAP1/027 group, and the dashed line represents the average OD_600_ of the non-BI/NAP1/027 group.

Variations in growth kinetics among different isolates may affect subsequent sporulation characteristics. To ensure that any future observations of sporulation rates were not a result of growth differences among strains, the change in optical density at 600nm (OD_600_) was measured over a 20 h time period. During the first 12 h there was no obvious difference in growth among *C. difficile* strains (data not shown). Therefore, based on the observed growth kinetics, we can exclude generic growth differences when interpreting subsequent sporulation data. Interestingly, after 20 h (Fig. 1) there was a significant difference in cell density among all strains of *C. difficile* (p<0.0001). In addition, the cell density at 20 h of the non-BI/NAP1/027 group was 24% higher than BI/NAP1/027 strains (p<0.0001). This finding is contrary to previous studies which, intriguingly, have reported that BI/NAP1/027 strains exhibited a higher cell density at 24 h than non-BI/NAP1/027 strains [9], [11].

### Ensuring minimal spore counts on inoculation of the sporulation medium

**Figure 2:**
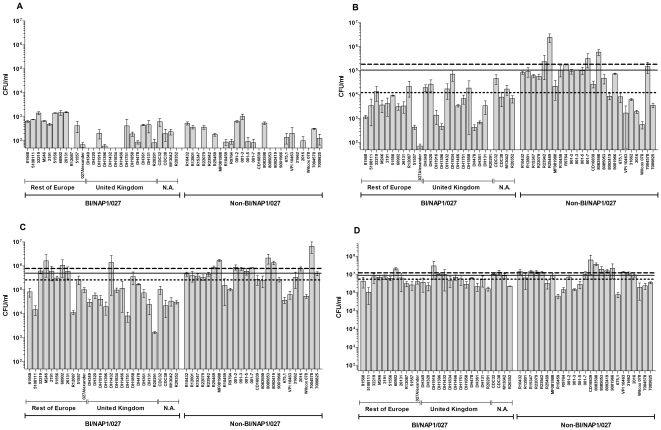
Development of heat-resistant CFU of 53 *C. difficile* strains. CFU following heat treatment (60°C for 25 min) were determined after (A) 0h; (B) 24h; (C) 48h; and (D) 120h incubation in BHIS broth. The BI/NAP1/027 strains were originally isolated from North America (N.A.), the UK, or the rest of Europe. The data represent the average of three independent experiments and error bars indicate the standard errors of the means. The solid line represents the average CFU/ml of all 53 isolates at 20h, the dotted line represents the average CFU/ml of the BI/NAP1/027 group, and the dashed line represents the average CFU/ml of the non-BI/NAP1/027 group. The detection limit for colony counts was 50 CFU/ml.

For studies of sporulation over a defined time-period (i.e. the rate of sporulation), it is crucial that the number of spores in the culture at 0 h are minimal, so as not to mis-interpret the subsequent spore counts. In preliminary work, we noted that five of the selected BI/NAP1/027 strains (DH478, DH1858, DH361, 027 Alexander and 51557) produced heat-resistant CFU counts in excess of 10^4^ CFU/ml (data not shown) at 0 h. Excessive carryover of spores from previous passages would suggest that sporulation has been initiated within the culture prior to the start of the experiment. This may in turn affect the rate of subsequent sporulation when compared to a culture where no spores are present at 0 h and could, consequently, lead to a bias among spore counts. Therefore, to ensure that as few spores as possible were present at 0 h, fresh stocks of the aforementioned strains were sub-cultured on rich medium supplemented with the bile salt taurocholate, to induce germination of any spores present in the culture [22], [23]. Following inoculation of the sporulation medium with these fresh cultures, fewer heat-resistant CFU were observed at 0 h (Fig. 2A) confirming that the high levels of heat-resistant CFU observed previously at 0 h were due to the presence of spores. This observation shows that an initial measurement at 0 h is essential to ensure that sporulation is observed only during the desired time period.

### Comparison of sporulation rates of BI/NAP1/027 and non-BI/NAP1/027 strains

The development of heat-resistant CFU was measured over a 120 h time period in order to understand the rate at which these isolates formed spores. Heat-resistant CFU were enumerated at 0 h, 24 h, 48 h and 120 h. A significant difference in the observed heat-resistant CFU was noted within the group of 53 strains at 24 h and 48 h (Fig. 2, panels B and C). After 24 h of growth, although the BI/NAP1/027 group appeared to form fewer heat-resistant CFU than the non-BI/NAP1/027 group the difference in heat-resistant CFU was not found to be statistically significant (p = 0.053). However, at 48 h the numbers of heat-resistant CFU were significantly higher in the non-BI/NAP1/027 group (p = 0.044). Interestingly, at 120 h there were no significant differences in the total amount of heat-resistant CFU observed among the group of 53 strains (Fig. 2D). Taken together, these data show that there is substantial variation in the rate at which different *C. difficile* strains form spores. However, this variation does not appear to be strictly associated with type and, most importantly, the rate of heat-resistant CFU development in BI/NAP1/027 strains does not appear to be higher than non-BI/NAP1/027 isolates.

**Figure 3:**
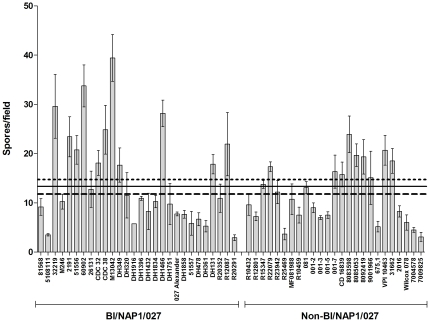
Total spore counts enumerated by phase-contrast microscopy after 120 h incubation in BHIS broth, from the same cultures used to measure heat-resistant CFU. The BI/NAP1/027 strains were originally isolated from North America (N.A.), the UK, or the rest of Europe. Spore titres were expressed as the number of spores per field of view. The data represent the average of three independent experiments and error bars indicate the standard error of the means. The solid line represents the average spore count of all 53 isolates at 20h, the dotted line represents the average spore count of the BI/NAP1/027 group, and the dashed line represents the average spore count of the non-BI/NAP1/027 group.

By definition, a heat-resistant CFU is a vegetative cell that has successfully completed sporulation, resisted the heat treatment, completed germination and, finally, returned to vegetative cell growth. Therefore, to accurately compare the number of spores independently of these other factors, one must enumerate spores using phase-contrast microscopy. To compare the spore titres among *C. difficile* strains, we enumerated spores of all 53 strains by microscopy, after 120 h of growth. Samples were taken from the same cultures used to measure heat-resistant CFU and the number of spores per field of view was counted at 1.5×40x magnification. We observed a significant difference in spore titre among the 53 strains (p<0.0001) but no significant difference (p = 0.18) was observed between the BI/NAP1/027 and non-BI/NAP1/027 groups (Fig. 3). These data show that although there is significant strain-to-strain variation in the total spore titres of different *C. difficile* strains, this variation does not appear to correlate to type. Furthermore, the BI/NAP1/027 strains did not have a higher sporulation capacity than the non-BI/NAP1/027 strains. It was intriguing that there was a statistical significance in the variation of spore titres among the 53 *C. difficile* isolates, as determined by microscopy, yet the corresponding variation in numbers of heat-resistant CFU at 120 h was not found to be statistically significant. The most obvious explanation for this finding is that the proportion of spores that form colonies may vary from strain to strain, a phenomenon that we have previously observed [16]. This suggests that differences may exist in spore heat resistance and/or germination characteristics among different *C. difficile* strains, which might be of interest for future studies.

### Diversity in sporulation rate within the BI/NAP1/027 type

Despite the continued use of small sample sizes, many previous studies have drawn general conclusions about the sporulation characteristics of the BI/NAP1/027 type. When comparing the numbers of heat-resistant CFU at 24 h, we observed significant diversity (p = 0.0009) within the BI/NAP1/027 group (Fig. 2B) although this did not correspond with the geographical regions of isolation. We noted significant differences in heat-resistant CFU observed at 24 h within the group of BI/NAP1/027 strains isolated in the United Kingdom (p = 0.032) but not within the group of BI/NAP1/027 strains isolated from the rest of Europe (p = 0.17). This indicates that even within a small geographical region such as the United Kingdom, substantial diversity can still exist between different BI/NAP1/027 strains, in terms of sporulation characteristics.

## Discussion

Spores are considered to be the vehicles of transmission of CDI [4]. Strains found to have an increased sporulation capacity are, therefore, of particular concern. In spite of our previous work suggesting that the variation in *C. difficile* sporulation rates is not associated with type [16], the suggestion that hypervirulent *C. difficile* types have a greater sporulation capacity than other types continues to circulate at international meetings, as well as in print [1], [10], [14], [18], [19], [20]. Here, we used an accurate, reproducible set of procedures to explore the sporulation properties of *C. difficile* isolates using the largest sample size to-date. Our comparison of 53 *C. difficile* isolates clearly indicates that neither the sporulation rate, nor the total sporulation capacity of the BI/NAP1/027 type were higher than that of non-BI/NAP1/027 strains. Indeed, our data suggest that the tested BI/NAP1/027 strains may form spores at a slower rate than the tested non-BI/NAP1/027 over 48 h.

We have previously reasoned that the current literature contains insufficient evidence to conclude that *C. difficile* BI/NAP1/027 strains exhibit an increased *in vitro* sporulation capacity [16], [17], [21]. When comparing the previous literature to the work from our laboratory, it is apparent that the different conclusions may reflect differences in the experimental approach. Importantly, when taking into account the different procedures used in other studies to measure sporulation, three previously published studies included the same *C. difficile* strain in their analysis, VPI 10463 [11], [13], [14]. These studies concluded that VPI 10463 is a low-sporulating strain, although both this study (Fig. 2 and Fig. 3) and our previous work are in disagreement with that conclusion [16]. These contradicting conclusions exemplify the pitfalls of undertaking studies using methods that cannot quantify sporulation independently of other important properties. Previously, we have introduced an experimental design that is capable of distinguishing between factors such as (i) vegetative cell growth; (ii) sporulation rate; (iii) total sporulation; (iv) the fate of non-sporulating vegetative cells; (v) spore stress resistance; and (vi) spore germination and outgrowth [16], [21]. By measuring sporulation over a period of at least 120 h it is possible to obtain an accurate account of the total number of spores produced and by taking measurements at multiple time-points, informed comments can be made on the rate of sporulation. Additionally, in order to make accurate statements relating to the absolute number of spores, exact spore titres must be enumerated using phase-contrast microscopy. Finally, suitable controls are essential to ensure that sporulation is observed only over the defined time-period and to ensure that the data are not inadvertently biased or skewed by technical errors.

The experiments conducted in this study underline the importance of ensuring minimal spore titres are present at the start of the assay. By measuring heat-resistant CFU at 0 h, we identified substantial carryover of spores into the sporulation medium in five *C. difficile* strains. Subsequent sub-culturing of these strains on appropriate media noticeably decreased the initial heat-resistant CFU count when the experiment was repeated. The development of spores in a liquid culture cannot be judged accurately without a measurement at 0 h as spores carried over from the starter culture may influence the rate of sporulation over a defined time period. Only one other study outside of our laboratory has included such a measurement of sporulation at 0 h [14], suggesting that some previous studies may not have analysed sporulation rates consistently within their sample of strains. In addition, no study outside of our laboratory has included a sporulation-negative control when measuring *C. difficile* sporulation rates. This is a fundamental control, as simple errors in experimental technique can lead to contamination of sporulation cultures which may affect subsequent spore counts. An ideal sporulation-negative control is a *spo0A* mutant, where the master regulator of sporulation has been inactivated, and this strain is available upon request from our laboratory [24]. Therefore, the lack of appropriate controls in a number of previous studies may perhaps make it difficult to validate the results [10], [11], [12], [13], [14], [15].

We also observed substantial diversity in sporulation rates within the BI/NAP1/027 type. For this reason, it seems obvious that all future *C. difficile* strain comparisons, not only those studying sporulation rates, should be based on as large a sample as possible. Furthermore, when describing a group of strains such as those belonging to the BI/NAP1/027 type, it seems advisable not to assume that the characteristics of one (or a small number of strains) is representative of all strains in that group. Indeed, recent evidence has suggested that patients infected with *C. difficile* BI/NAP1/027 were not any more likely to develop “severe” disease than patients infected with other PCR-ribotypes [25]. Therefore, we propose that while some BI/NAP1/027 strains may cause a more severe disease, this may not be the case for all strains of the BI/NAP1/027 type. To the contrary, individual *C. difficile* strains belonging to other types may well be as virulent as BI/NAP1/027 strains.

Further studies of *C. difficile* sporulation diversity are clearly needed. Such studies could examine sporulation efficiency by measuring the proportion of vegetative cells that form spores. Additionally, by observing the numbers of vegetative cells in a sporulating culture an argument could then be made regarding the fate of vegetative cells that do not enter the sporulation process. The obvious question then still remains of how the *in vitro* sporulation characteristics described in this study relate to sporulation proficiency *in vivo*. Consequently, until our understanding of *C. difficile* sporulation and germination *in vivo* improves, it will be difficult to associate the *in vitro* characteristics observed in this study with disease severity.

The emergence of some *C. difficile* BI/NAP1/027 strains associated with increased disease severity provides continuing challenges in the healthcare setting. Understanding how sporulation rates vary among clinical isolates of *C. difficile* is a small but essential step to understanding how emerging, hypervirulent *C. difficile* types differ from those which are less frequently associated with outbreaks of severe disease. Based upon the data presented in this manuscript and the limitations of some previous studies, it is possible that current research directions associated with the sporulation mechanisms of *C. difficile* may be based on incorrect interpretations of preliminary data.

## Materials and Methods

### Bacterial strains and culture conditions

A total of 28 BI/NAP1/027 and 25 non-BI/NAP1/027 strains of *C. difficile* were chosen for analysis (Table 1). The BI/NAP1/027 group included isolates from the United Kingdom (n = 13), mainland Europe (n = 11) and North America (n = 4). The non-BI/NAP1/027 group included strains isolated in Europe belonging to PCR-ribotypes 001 (n = 4), 002 (n = 6), 003 (n = 1), 017 (n = 1), 078 (n = 5), 081 (n = 1) and 106 (n = 7).

*C. difficile* strains were grown at 37°C in an anaerobic workstation (Don Whitley, United Kingdom) in BHIS (brain heart infusion supplemented with yeast extract [5 mg/ml, Oxoid] and L-cysteine [0.1%, Sigma, United Kingdom]) broth or agar, in which *C. difficile* sporulates efficiently [23].

### Preparation of C. difficile spores

*C. difficile* strains were selected on BHIS agar supplemented with cefoxitin (8 µg/ml) and cycloserine (250 µg/ml). Sporulation of *C. difficile* was achieved through incubation of cultures in BHIS broth in anaerobic conditions at 37°C for 5 days. Overnight cultures of *C. difficile* isolates in BHIS broth were used to inoculate a starter culture in BHIS broth 1 in 100, which was grown to an OD_600_ of between 0.2 and 0.5 to ensure the lack of spores on inoculation of the sporulation medium. The sporulation medium was then inoculated 1 in 100 with the starter culture.

### Measurement of sporulation rates

In order to measure colony formation, 500 µl samples of the sporulation medium were removed from the anaerobic chamber and heated at 60°C for 25 min to kill vegetative cells but not spores. Samples were then returned to the anaerobic chamber, serially diluted in phosphate buffer saline and plated onto BHIS agar supplemented with the bile salt taurocholate (0.1%, Sigma, United Kingdom) to induce germination and enhance recovery of *C. difficile* spores [22], [23]. Plates were incubated for 24 h before CFU were enumerated. For all measurements of heat-resistant CFU, a *spo0A* sporulation-negative control was included to rule out technical errors [24].

### Measurement of total sporulation capacities

Total sporulation after 5 days incubation in BHIS was assayed by counting spores under phase contrast microscopy. After 120 h growth, a 10 µL aliquot of the sporulation medium was loaded onto a microscopy slide, air dried, and visualised at 1.5×40x magnification. Sporulation was expressed as the number of spores counted per field of view and a minimum of four fields of view were counted for each replicate. Spores were enumerated under live fields of view to allow for continuous z-plane adjustments and, therefore, to ensure that all phase-bright spores in the field were identified and counted.

### Biological replicates and statistical analyses

All data presented in this manuscript represent the results of three independent experiments. All statistical analysis was carried out in GraphPad Prism using Student's t-test for comparison of the BI/NAP1/027 group with the non-BI/NAP1/027 group and one-way analysis of variance with Tukey's *post hoc* compensation for multiple comparisons of individual strains.
